# Supraspan memory performance is impaired in subjective cognitive impairment compared to cognitively unimpaired individuals

**DOI:** 10.1038/s41598-025-07664-5

**Published:** 2025-07-02

**Authors:** Ove Almkvist, Måns Gyllenhammar, Sofia Norberg, Mia Lu Roeseler, Eric Westman, Urban Ekman

**Affiliations:** 1 Division of Clinical Geriatrics, Department of Neurobiology Care Sciences and Society, Karolinska Institutet, Neo Building Floor 7, 14157 Stockholm, Sweden; 2 Theme Inflammation and Aging, Karolinska University Hospital, Stockholm, Sweden; 3 Department of Psychology, Stockholm University, Stockholm, Sweden; 4 Medical Unit Allied Health Professionals Women’s Health and Allied Health Professionals Theme, Karolinska University Hospital, Stockholm, Sweden; 5 Department of Neuroimaging, Centre for Neuroimaging Sciences, Institute of Psychiatry, Psychology and Neuroscience, King’s College, London, UK

**Keywords:** Subspan, Supraspan, Memory, Subjective cognitive impairment, Psychology, Diseases

## Abstract

The objective cognitive status is incompletely known in Subjective Cognitive Impairment (SCI) and similar constructs. To characterize subspan and supraspan memory performance in groups with SCI, Mild Cognitive Impairment (MCI), and Cognitively Unimpaired individuals (CU) in relation to brain atrophy and CSF biomarkers. Performance on subspan (Digit Span Forward) and supraspan memory (trial 1 in the Rey Auditory Verbal Learning test) was investigated in patients diagnosed with SCI (n = 237) and MCI (n = 1038) from memory clinics in the Stockholm region and CU individuals from Stockholm university, Department of Psychology (n = 124). All participants had an extensive cognitive assessment. In addition, MCI and SCI patients had a comprehensive clinical examination, brain imaging scan (medial temporal lobe, cortical global atrophy, and white-matter-hyperintensities), and CSF biomarker analyses (Abeta, p-tau, and total-tau). Groups differed significantly in demographic characteristics, which were adjusted for in all analyses. The three groups differed significantly on supraspan, but not subspan, memory performance in line with the severity of cognitive impairment. The test-by-group interaction was also significant showing that SCI was characterized by selective supraspan impairment in conjunction with memory overload and in contrast to the previous conception of no objective impairment in SCI. CSF biomarkers and brain abnormality in MTA, GCA, and WMH did not provide additional predictive power over the diagnostic group on supraspan in SCI. Supraspan memory performance differed by diagnostic group in relation to the degree of cognitive impairment in memory clinic participants (CU > SCI > MCI) indicating objective memory impairment in SCI interpreted as due to supraspan overload.

**Supplementary Information:**

The online version contains supplementary material available at 10.1038/s41598-025-07664-5.

## Introduction

The construct of Subjective Cognitive Decline (SCD) describes individuals who report persistent subjective cognitive symptoms that have not existed previously and do not show any objectively measured cognitive impairment^1–4^. The prevalence of SCD in the aging population has been estimated to be a quarter of individuals over 60 years of age^5^. The most frequently reported symptom is the inception of memory complaints^6^. Findings show that SCD individuals are at a higher risk to develop future cognitive impairment e.g., Mild Cognitive Impairment (MCI) and dementia such as Alzheimer’s disease, compared to individuals with no cognitive complaints^7–13^. Further, abnormalities in SCD have been reported in white matter with brain imaging^14–16^, CSF biomarkers^17,18^, PET biomarkers^8,19^. In addition, low cognitive performance has been found in SCD, although within the normal range^8,20^ that were not identified in baseline examinations. In addition, affective symptoms such as depression and anxiety have been described in previous reviews of SCD^9,21,22^. In agreement with these findings, self-reported concern about cognitive change has been added to the revised criteria for SCD together with seeking medical help as a measure of coping^2^. Together, these findings indicate that SCD may represent a pathologic stage that occurs earlier than MCI but is not associated with any marked objective cognitive changes. In contrast, there are studies suggesting that the majority of SCD patients do not develop dementia or MCI over six years of follow-up making SCD a predominantly benign condition^23^.

Recent research has shown that at least some individuals diagnosed with SCD may have subtle cognitive impairments that could predict future progression to MCI years later^7,24^, although the type of cognitive dysfunction is unsettled. In one study, the total learning score (sum of correct responses over trials) was most associated with amnestic MCI, and the trial 1 score on the RAVL test^25,26^ was most associated with non-amnestic MCI, while predictive power was lacking in other cognitive functions (global cognition, language, visuospatial, short-term memory, and processing), when demographic characteristics were accounted for^7^. Another study^24^ showed that progression was predicted by age, memory, and processing speed. Furthermore, recently we showed that individuals diagnosed with subjective cognitive impairment (from now on, SCI is handled as comparable to SCD) performed significantly poorer than the cognitively unimpaired adults (CU) on trial 1 in the Rey Auditory Verbal Learning test (RAVL;^27^). It is well known that performance in tasks with a limited number of items (subspan) and tasks with an extended number of items (supraspan) are associated with different mental processes and different regional brain involvement^28–30^. Subspan performance involves apprehension, attention, temporary storage as well as manipulation of the temporarily stored information that is known to activate prefrontal regions and parietal cortex^30–33^. Supraspan performance involves processes of encoding/learning and activation of brain structures in the medial temporal lobe^29,30,34^.

Hypothetically, the difference in performance relates to difference in task demands in subspan and supraspan particularly due to the amount of information to process, i.e., limited in subspan (less than the capacity limit) and extended in supraspan (15 items). The number of items in the classical subspan task is known as the “Magical number” corresponding to 7 ± 2 items^35^, whereas the number of items in supraspan tasks exceeds the capacity of the subspan task. This difference between subspan and supraspan has been linked to the memory load concept^29,36^. In a study of memory overload using the n-back task (4 items vs. 2 items), results showed increased dorsolateral prefrontal cortex activity linked to overload and impaired memory performance, and increased amygdala activity as a possible emotional reaction following difficulties and failure due to the memory overload^36,37^.

The challenge remains to properly characterize the three groups (CU, SCI and MCI). Hypothetically, there is no marked cognitive or subjective impairment or change in CU (see^38^, while there are both subjective and observable cognitive changes in MCI^39^. The question is whether SCI should be understood solely as self-experienced cognitive impairment or more critically as affected by objective cognitive change as well?

The purpose of the present study was to investigate the interaction between performance in two memory tasks, subspan vs. supraspan, and three groups varying in degree of cognitive impairment (MCI, SCI, and CU). The subspan task was operationalized by the Digit Span Forward test (DSpF) and the supraspan task was operationalized by trial 1 in the RAVL test (t1 RAVL). Characterization of MCI and SCI groups was extended by using information from brain imaging and CSF biomarkers. Based on findings in previous studies^7,24,27^, the hypothesis was that there is a task-by-group interaction as shown by increasing group differences (MCI > SCI > CU) on the supraspan task in contrast to relatively similar performance across groups in the subspan test. Hypothetically, the difference between performance in the subspan and supraspan tasks could be supported by differences in brain atrophy (more prominent in supraspan than subspan), primarily in MTA^31,34^ as well as in CSF biomarkers (more prominent in supraspan than subspan). This hypothesis deviates from the common understanding that SCI and CU are cognitively comparable^1,2,4^.

## Methods

### Participants

Patients diagnosed with SCI (n = 237) and MCI (n = 1038) were recruited from the MemClin project collecting data from 9 of 10 memory clinics in the Stockholm metropolitan area^40^ between April 2016 and January 2021. The MemClin project has an unselected approach, and all patients referred to a neuropsychological examination were considered for participation. Completeness of collected data varied between sites, most likely because of variations in examination methods used on a patient-to-patient basis.

In addition, CU individuals (n = 124) were volunteers examined by students practicing psychological testing at the Department of Psychology, Stockholm University. They were not paid for participation but offered information about their results and an evaluation of their cognitive status. Among the original 146 volunteers, 11 were excluded due to two or more missing test results, and 11 were excluded due to two or more test results < −2 SD below the age-related mean. All participants were 65–90 years of age, fluent in Swedish, and evaluated as CU.

### Clinical examination and diagnosis

Details of the clinical examination of patients have been described previously^40^. In brief, diagnosis was determined at each Memory clinic through a multidisciplinary consensus meeting based on all information from the clinical examinations. The SCI patients did not have any objectively verified cognitive impairment based on the clinical assessment in accordance with ICD-10 diagnostic criteria^41^, while the presence of symptoms was self-reported or reported by a close informant^1,2,4,9^. The MCI patients were diagnosed according to international consensus criteria^39^ including subjective symptoms as well as objective findings of cognitive decline that did not fulfill the criteria for dementia^41^.

The examination included structural brain imaging (derived from computer tomography and/or magnetic resonance imaging) of medial temporal atrophy (MTA;^42^), global cortical atrophy (GCA;^43^), and white matter hyperintensities (WMH;^44^).

CSF biomarkers were analysed with standard CSF Abeta-sensitive methods (ELISA, CLEIA, or ECLIA) that are highly concordant^45^. This result supports that the same cut-off values were used for all participants. The cut-off for Abeta abnormality in the present study was ≤ 550 ng/l. For p-tau and total-tau, the cut-off values of abnormality were p-tau ≥ 80 ng/l, and t-tau ≥ 400 ng/l^40^.

The volunteers from Stockholm University were evaluated as cognitively unimpaired (CU) according to a comprehensive examination including 10 tests from WAIS-IV^46^ and seven non-WAIS tests, the same tests as used in the MemClin examination (see the Supplement for a detailed description of test results (M ± SD) for the three groups).

### Memory tests

Two specific memory tests were used to assess subspan memory (Digit Span Forward, DSpF;^46^) and supraspan memory (trial 1 in the Rey Auditory Verbal Learning test, RAVL;^25,26^). The items are presented orally by the test administrator and responses are delivered orally by the subject in both tests. However, there are important differences in the procedure in the subspan and supraspan tests. The subject should report items (digits) in the same order as they were presented in DSpF, while the items (words) should be reported in any order in RAVL trial 1. The number of correct responses was used as the outcome measure in both tests. The maximum number of items to be remembered in the subspan task is commonly nine or less in accordance with the capacity limit of short-term memory. Furthermore, the procedure of presentation differs considerably between tests. In the subspan task, there is a series of presentations starting with 3 digits, and if they are correct, the presentation continues with 4 digits, and so on until the subject fails in two subsequent trials with the same number of items. In contrast, the number of items to be remembered in the supraspan task is 15 and they are presented once, without any pretest exercise. In both tests, the instructions make it clear what kind of performance is required. Previous research has demonstrated that the use of digits vs words in the subspan has a minimal influence on the results^47^.

As previously mentioned, response items need to be delivered in correct order in DSpF but not in t1 RAVL. Despite this, the subject commonly starts reporting some of the recently presented words (recency phenomenon), followed by some words that were first presented (primacy phenomena). These phenomena may indicate that recency represents a readout of acoustic short-term memory. Hypothetically, this same mental process (readout) is used during the DSpF testing. In contrast, primacy represents the binding of items to a timeline or sequence reflecting conscious efforts to encode the material. The two suggested mental processes illustrate hypothetical events that differentiate subspan and supraspan memory performance^29,30^.

### Ethical approval

All patients had signed consent to use clinical data for research which was approved by the Regional Ethics Committee in Stockholm (Dnr 2016/29-31/1). A similar document for CU was approved by the Regional Ethics Committee in Stockholm (Dnr 2017/549-31/1). All collected data were handled according to the EU General Data Protective Regulation and applicable Swedish legislation to ensure patient security. The study was performed in agreement with the Declaration of Helsinki rules.

### Statistical analyses

**Fig. 1:**
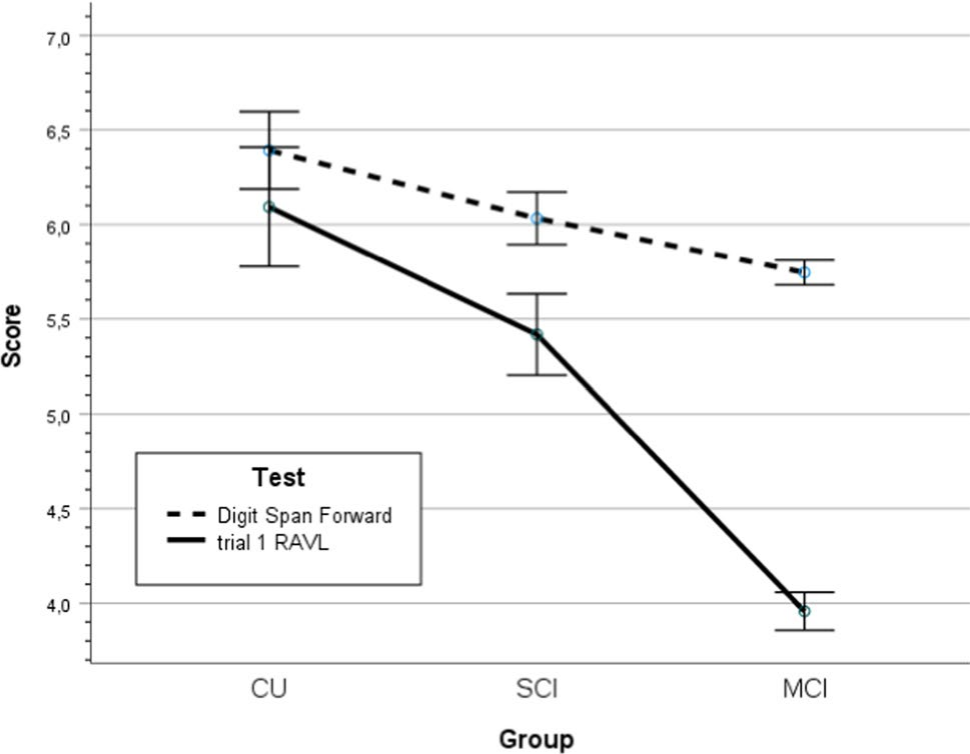
Score on tests of subspan (Digit Span Forward) and supraspan (RAVL trial 1) memory with 95% confidence interval for cognitively unimpaired individuals, subjective cognitive impairment and Mild Cognitive Impairment (CU, SCI, and MCI).

Calculations were carried out by using SPSS, version 28. One-way (diagnostic group) ANOVA was used to analyse group differences in demographics, global cognition (MMSE;^48^), and subspan and supraspan memory tests (see Tables 1 and 2 and Fig. 1) showing descriptive data, significance, and effect size (η^2^). A two-way repeated measures (tests) MANCOVA with the diagnostic group as independent variable and demographics as covariates were used to analyse the interaction between the tests (subspan and supraspan) vs group and brain abnormality (MTA, GCA, and WMH defined as absent/very mild, mild, and moderate/severe, separately for each brain measure, see Table 3 and Table 1 in the Supplement for statistical analyses. Post-hoc t-test were used to delineate the difference between groups and tests. The importance of CSF biomarkers (Abeta, p-tau and total-tau) was analysed separately for each biomarker using MANCOVA with tests as DV, diagnostic groups, and CSF abnormality in two categories (defined as abnormal or normal) as IVs and demographics as covariates, see Table 4 and Supplement Table 2.

## Results

The demographic characteristics (age, sex, and education) and global cognitive function as indicated by the MMSE^48^ for the three groups are presented in Table 1. The groups differed significantly in all demographic characteristics (age, sex distribution and years of education) and global cognitive function (MMSE). Post-hoc pairwise group differences (See Table 1) showed that MCI patients were significantly older than SCI and that SCI patients were significantly older than CU. Female frequency was significantly higher in MCI than SCI, but not significantly different in SCI vs. CU. Pairwise post-hoc group difference in education showed that SCI patients were significantly more educated than MCI, but not in CU vs. SCI.

**Table 1:** Demographic (age, sex, and years of education) and background clinical characteristics (MMSE) in MCI, SCI, and CU groups and two-way (diagnostic group) Outcome of ANOVA’s on demographics (age, sex and education) and MMSE with *p*-values and effect size (*η*^*2*^) as well as post-hoc pair-wise differences in age, female frequency (%), years of education and MMSE.

	MCI	*p*	SCI	*p*	CU	*p*	*η*^2^
N (% females)	1038 (53%)	**	237 (42%)	^ns^	124 (40%)	< .001	.010
Age, y	76.7 ± 6.4	***	73.7 ± 7.8	**	71.6 ± 5.4	< .001	.064
Education, y	13.2 ± 3.5	**	13.9 ± 3.7	^ns^	14.2 ± 2.4	< .001	.010
MMSE, score	27.4 ± 2.0	***	28.8 ± 1.6		–		.714

* = *p* < .05, ** = *p* < .01, ***p =  < .001, ns = not significant.

### Subspan and supraspan memory in MCI, SCI and CU

Subspan and supraspan test results for the three groups (MCI, SCI, and CU) are presented in Table 2. The main effects of group and demographic characteristics were significant as well (*p*’s < .001; group, age, sex and education, respectively). Correcting for demographic factors, there was a significant test-by-group interaction in line with the hypothesis (*p* < .001, *η*^2^ = .079), see Fig. 1. In addition, the test-by-age and test-by-sex interactions were significant (*p*’s < .001, *η*^2^ = .034, *η*^2^ = .024, respectively), but not test-by-education. The pairwise subgroup differences between MCI vs SCI and SCI vs CU were significant (*p*’s < .01, Cohen’s *d* > .280). The pairwise test difference between DSpF and RAVL t1 were not significant in CU (*p* > .1, *d* = 0.004), but significant in SCI (*p* < .001, *d* = .231) and MCI (*p* < .001, *d* = .1.006), see Table 2 and Fig. 1.

**Table 2:** Test results in subspan (Digit Span Forward) and supraspan (RAVL trial 1) for MCI, SCI and CU groups with one-way (group) ANOVA statistics (p-value and effect size, η^2^) and covariates (age, sex and education) with p-values as well as p-value for test difference within each group.

Test	MCI		SCI		CU		*η*^2^	A	S	E
Raw score	n = 1022	*p*	n = 230	*p*	n = 124	*p*				
DSpF	5.74 ± 1.06	**	6.04 ± 1.09	**	6.40 ± 1.10	< .001	.028	***	***	***
*p* (test)	**		**		ns					
RAVL t1	3.89 ± 1.64 **		5.58 ± .72	**	6.39 ± 2.00	< .001	.197	***	***	***

A = Age, S = Sex, E = education.

* = *p* < .05, ** = *p* < .01, ***p < .001, ns = not significant.

### Brain atrophy in SCI and MCI

Brain atrophy was investigated in about 81% of all patients (both in SCI and MCI), see Table 3. The frequency of brain abnormality (binarized as 0 = absent or very mild vs 1 = all other categories) was significantly different between groups on MTA (*p* < .001, *d* = .512), GCA (*p* < .05, *d* = .289) and WMH (*p* < .001, *d* = .315)..

**Table 3:** Frequency and percentage of brain abnormality in Medial Temporal Atrophy (MTA), Global Cortical Atrophy (GCA) and White Matter Hyperintensity (WMH) for SCI and MCI patients.

Scale	MTA		GCA		WMH	
	SCI n, %	MCI n, %	SCI n, %	MCI n, %	SCI n, %	MCI n, %
0 and 0.5	62, 33%	146, 17%	46, 23%	142, 17%	77, 41%	233, 28%
1 and 1.5	90, 48%	356, 42%	124, 62%	476, 56%	69, 37%	326, 39%
2 and more	38, 20%	348, 41%	30, 15%	233, 27%	42, 22%	283, 34%
Σ	190	850	200	851	188	842

Absent or very mild atrophy (0 and 0.5), mild (1 or 1.5) or marked/severe (2+).

### The impact of group (SCI and MCI) and brain atrophy (MTA, GCA and WMH) on subspan and supraspan memory tests

The MANCOVA analyses on the test (subspan and supraspan) as repeated dependent variable and brain atrophy and group as independent variables showed that the effect of tests was significant (*p* < .05, *η*^2^ = .008), see Table 2 in the Supplement for details of the statistical analysis. The effect of diagnosis was significant (*p* < .001, *η*^2^ = .103), but not the effect of MTA abnormality. The test-by-group interaction was significant (*p* < .001, *η*^2^ = .043), but not the group-by-MTA abnormality, the test-by-MTA, and three-way interactions. The interactions for test-by-age and test-by-sex were significant (*p*’s < .001, *η*^2^ = .031, *η*^2^ = .027, respectively), but not the test-by-education. The main effects of age and sex were significant (*p*’s < .001, *η*^2^ = .015, *η*^2^ = .010, respectively), but not education.

The pattern of MANCOVA results with WMH abnormality was equivalent to the pattern of significance and non-significance results for MTA. The same outcome pattern was obtained for MANCOVA with GCA with one exception, the test-by-group interaction was significant (*p* < .001, *η*^2^ = .033).

In summary, the MANCOVA results on subspan and supraspan memory tests as dependent variables vs diagnostic group and brain atrophy (MTA, GCA and WMH) as IVs and demographics as covariates showed that brain atrophy MTA and WMH) did not add predictive power to the analyses of test performance over the diagnostic group, while GCA influenced test performance significantly (*p* < .05, *η*^2^ = .004). There was a general influence by age, sex, and years of education on test performance (*p*’s < .01, *η*^2^ = .015, *η*^2^ = .007, *η*^2^ = .030, respectively) as well as an interaction with age and sex on test performance (*p*’s < .001, *η*^2^ = .030, *η*^2^ = .026, respectively).

### CSF biomarkers in SCI and MCI

The CSF biomarkers were divided into categories of normal or abnormal memory values using the clinical cut-off values, see the Method section. In total, CSF examination was performed in 42% of all patients (n = 530) and more frequently in SCI (55%) than in MCI (32%), see Table 4. The frequency of abnormal results was significantly different between diagnostic groups on all three biomarkers, clearly higher in MCI than in SCI for all three biomarkers (*p*’s < .001, x), see Table 3 in the Supplement. The highest frequency of abnormal CSF values was observed in total-tau (47%) for MCI) vs 26% for SCI. In Abeta, the abnormal values were found in 32% of MCI vs 13% in SCI and still lower abnormality frequencies were observed in p-tau, 24% for MCI compared to 4% in SCI.

**Table 4:** Frequency of CSF sampling in SCI and MCI patients (%) and frequency of abnormal (%) and normal CSF biomarkers (Abeta, p-tau, and total-tau).

Measure	SCI n = 90		MCI n = 440	
	Normal	Abnormal	Normal	Abnormal
	n, %	n, %	n, %	n, %
Abeta	78, 87%	12, 13%	301, 68%	139, 32%
p-tau	86, 96%	4, 4%	334, 76%	105, 24%
Total-tau	67, 74%	23, 26%	233, 53%	206, 47%

### The impact of group (SCI and MCI) and CSF biomarkers (Abeta, p-tau and total-tau) on subspan and supraspan memory tests

The possible influence of CSF biomarkers on the test-by-group interaction was investigated in the cohort, in which a CSF examination was performed, i.e., n = 530 of the original cohort of MCI and SCI patients (n = 1274). The two cohorts, with and without a CSF examination, were not comparable, because those with a CSF examination were significantly younger (*p* < .001, *d* = .26) and performed better in RAVL learning (*p* < .05, *d* = .14) than those without a CSF examination, while the proportion of SCI and MCI was not significantly different (*p* > 0.1).

### CSF Abeta

**Fig. 2:**
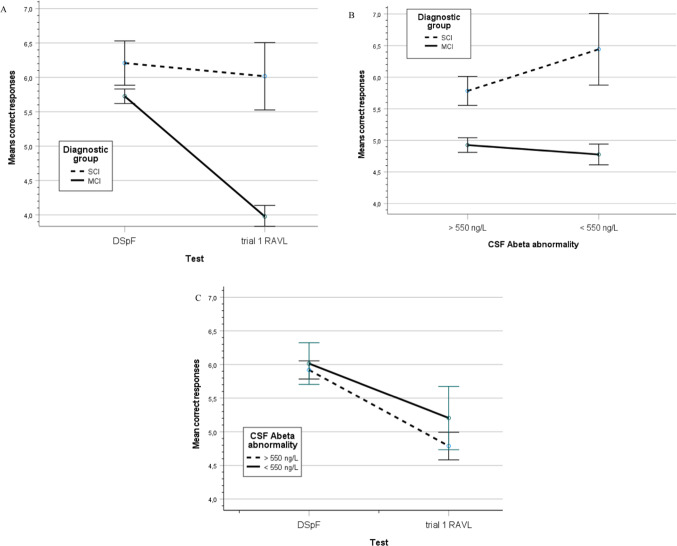
(**A**) A line graph with 95% CI showing the significant diagnosis-by-CSF Abeta interaction across tests. (**B**) A line graph with 95% CI showing the significant diagnosis-by-test interaction across CSF Abeta abnormality. (**C**) A line graph with 95% CI showing the non-significant CSF Abeta-by-test interaction across diagnostic groups.

To analyze the influence of diagnostic group (MCI and SCI) and Abeta pathology on memory load, a two-way MANCOVA with tests (DSpF and t1 RAVL) as repeated dependent variable and group and CSF Abeta abnormality (yes/no, defined by cut-off data, see the Method section) as independent variables and demographics as covariates (age, sex and education). For details of the statistical analysis, see Table 3 in the Supplement. The influence on tests was not significant. The effect of group was markedly significant (*p* < .001, *η*^2^ = .087), but not the influence of CSF Abeta abnormality. Among the six two-way interactions, four were significant: the test-by-group (*p* < .05, *η*^2^ = .050; see Fig. 2A), Abeta-by-group (*p* < .01, *η*^2^ = .011; see Fig. 2B) showing better test results in SCI with Abeta abnormality, test-by-age (*p* < .001, *η*^2^ = .031) and test-by-sex (*p* < .001, *η*^2^ = .027), but not the test-by-education and test-by-Abeta pathology, despite the visual impression, see Fig. 2C. In Fig. 2C, the two tests were collapsed. If tests are separated, the result showed that SCI and MCI patients performed similarly in DSpF and independent of Abeta level, but differently in the trial 1 RAVL test and better, when Abeta was abnormal compared to normal Abeta (see comment in the discussion). However, this three-way (test-by-group-by-Abeta pathology) was not significant.

### CSF p-tau

The pattern of significant results with MANCOVA using CSF p-tau pathology as biomarker together diagnostic group as independent variables and demographic covariates on the two memory tests as repeated dependent variable was the same as obtained with CSF Abeta. The only exception was a non-significant outcome for group-by-CSF p-tau interaction (*p* > .1) in contrast to the significant group-by-CSF Abeta interaction, see above.

### CSF total-tau

The pattern of significant results with CSF total-tau pathology showed a pattern of results that was in correspondence with the results for CSF p-tau pathology.

In summary, the MANCOVA results on subspan and supraspan memory tests as dependent variables vs diagnostic group and CSF biomarkers (Abeta, p-tau and total-tau) as IVs and demographics as covariates showed that no single CSF biomarkers did add predictive power to the analyses of test performance over the diagnostic group, while the Abeta-by-group was significant. There was a general influence by age, sex, and years of education on test performance.

## Discussion

### The main finding

In keeping with the hypothesis, a significant group-by-test interaction was shown by a minor decrease across groups in subspan memory from CU to SCI to MCI and a major decrease across groups in supraspan memory from CU to SCI to MCI. Furthermore, the difference between subspan and supraspan memory was not significant in CU, but significant in SCI, and markedly significant in MCI. In contrast, the difference between groups on supraspan memory was significant in all three groups, largest in MCI weaker in SCI, and least in CU. This pattern of results supports the conclusion that there is a clear objective memory impairment in SCI in contrast to the current understanding that SCI and SCD do not present any cognitive decline^1,2,4^. The subspan vs supraspan difference has also been shown previously in AD^47^ and has been interpreted as caused by memory overload^29^. To our awareness, the supraspan memory impairment in SCI is a novel finding.

A possible explanation for the unexpected objective memory impairment is that SCI patients may be misdiagnosed and that they should be diagnosed as part of the MCI diagnosis as has been suggested previously^7,8,49^.

Another possible interpretation of objective memory impairment in SCI is to introduce two types of SCI, stable and declining^20^. It is possible to use brain imaging or CSF biomarker or cognitive methods to predict future dysfunction in SCI^8,16,17,20,50^.

A third interpretation is to relate the findings to variation in memory load, i.e., number of items that are presented in the two tests. Hypothetically, the low-load test (DSpF, subspan) involves at least two requires mental processes, attention and memory. The DSpF test is a manageable task, and the performance is successful until the individual reaches the short-term memory capacity limit^35^. This task relies on activation of brain regions particularly in the dorsolateral prefrontal cortex and bilateral inferior parietal lobule^31,32,51^. In addition, the performance in the DSpF test is to some degree related to attention and linked to alertness, orientation and control^51,52^, that is associated with brain activity in attentional brain networks^51,52^.

In contrast, the high-load test (t1 RAVL, supraspan) is not manageable, the individual will fail, because the number of items to report are too many. During the presentation of the 15 words, the time is limited for elaborating and learning the material. The activation of brain regions that are important for learning, the medial temporal region and particularly hippocampus^29,30,34^ fall short. The individual may be overwhelmed and experience emotional discomfort due to mastering difficulties. How the individual tolerates stress will be important for optimal performance. To speculate this factor explains that the result on supraspan is lower than the result on subspan in majority of SCI individuals.

Interestingly, a fMRI study on working memory overload in healthy young adults showed lower performance with n-back test (4 vs 2 items) in conjunction with brain activation in dorsolateral prefrontal cortex and amygdala activation in line with failure degree^36^. In this study, the participants were evaluated as healthy, and the activation of amygdala was a direct reaction to the difficulty in the n-back test. In previous research it has been shown that personality features of anxiety and stress susceptibility are more frequent in SCI compared to healthy controls^53^. Here it must be pointed out that there was no assessment of emotional reactions during testing or evaluation of the individual’s status in terms of anxiety or other psychic features. So, it is an open question whether the result on supraspan is direct a reaction to the test difficulty or if it represents a proneness in general to be tested.

Now, worries in general have been added to the revised criteria of SCD^2,9,21,22,49^. To speculate, a comparison of performance in tests varying in load may be a more sensitive method for detecting subtle cognitive impairment compared to using standard cognitive tests in clinical assessment^54^. Indeed, it is a general dilemma to differentiate normal functions and the first early changes in preclinical disease like SCI^55,56^.

### The second finding

Brain atrophy did not add predictive power to the association between diagnostic group and memory test performance. This was unexpected because memory performance is typically associated with brain atrophy, particularly in MTA^30,34^. However, the influence of brain atrophy was probably discounted by using diagnostic group as the primary predictor. Furthermore, non-optimal conditions with reduced variation (low frequency of clear abnormal atrophy) or too large and unspecific brain regions may limit the sensitivity of brain imaging in the present study.

### The third finding

The CSF pathology (Abeta, p-tau and total-tau) did not have a significant effect on memory test performance as a single factor independent of group. However, the interaction between tests and diagnostic group was significant showing relatively good performance for both groups on the subspan test and better performance in SCI in the supraspan test (see Fig. 2A). The group-by-Abeta abnormality interaction was significant (Fig. 2B). According to visual inspection, the MCI group performed poorly and independently of Abeta levels, while SCI patients performed better when they had abnormal Abeta (n = 12) compared to normal values (n = 78). However, this 3-way interaction was not significant. Presently, the favorable Abeta result on supraspan is considered a spurious result rather than reflecting a valid finding.

The clinical diagnosis tied to severity of disease was the main factor that influenced the memory test results, while there was no significant influence on the memory tests by CSF p-tau or total-tau. The main limiting factor for CSF biomarker influence was that less than half of the participants had an examination of SCF biomarkers. However, subgroups with vs without CSF data were comparable in comparable memory performance.

### Strength and shortcomings

The relatively large sample size and the naturalistic character of the cohort recruited from memory clinics in the Stockholm metropolitan region represent favorable features of the study. However, the clinical diagnosis of patients may include several etiologies because there was a lack of extensive biological data to support the diagnostic specificity. In the same vein, there was a lack of longitudinal data to help diagnose patients as SCI and MCI. Further, it was a drawback that data were missing particularly for CSF biomarkers and to some extent for brain imaging. The clinical examination did not separate self- and informant-reports on symptoms. There was no assessment of possible emotional reactions in relation to the memory tests; furthermore, there was no assessment of neuropsychiatric proneness in participants. Therefore, the results of this study need to be replicated.

### Implications

The present study was based on the idea that cognitive load in memory indicated by number of items was an important factor in contrast the standard test idea based on task difficulty (cf test design). Interestingly, the idea on load is used in the subspan task to examine the memory span. In a similar vein the number of items is an important factor for perception (cf. the concept subitizing) and practicing mental search (cf. xx). In this study, the idea of cognitive load had clinical implication for diagnosis of stages of cognitive impairment (CU vs SCD vs MCI). A second implication relates to the possible interaction between cognitive performance and the impact of emotional reactions that may disturb cognitive performance. This hypothesis has to be investigated in future research.

## Conclusion

SCI was characterized by objective memory impairment in high-load supraspan memory test compared to CU individuals. This is a novel finding with possible clinical prospects. The effect was interpreted as selective emotional involvement due to memory overload in supraspan performance in SCI. In the low-load subspan memory test, the diagnostic groups performed equally. The main explanatory factor for memory performance was load-dependent and clinical group that included CSF abnormality (Abeta, p-tau and total-tau) and brain atrophy (MTA, GCA and WMH).

## Electronic supplementary material

Below is the link to the electronic supplementary material.

Supplementary Material 1

Supplementary Material 2

Supplementary Material 3

## Data Availability

Data availability statement The data may be available upon reasonable request from Associate Professor Urban Ekman, urban.ekman@ki.se.

## References

[1] Jessen, F. et al. A conceptual framework for research on subjective cognitive decline in preclinical Alzheimer’s disease. *Alzheimers Dementia.* **10**, 844–852 (2014). 10.1016/j.jalz.2014.01.001 PMC4317324 24798886

[2] Jessen, F. et al. The characterization of subjective cognitive decline. *Lancet Neurol.* **19**, 271–278 (2020). 31958406 10.1016/S1474-4422(19)30368-0 PMC7062546

[3] Molinuevo, J. L. et al. Subjective cognitive decline initiative (SCD-I) working group. Implementation of subjective cognitive decline criteria in research studies. *Alzheimers Dement.* **13**, 296–311 (2017). 27825022 10.1016/j.jalz.2016.09.012 PMC5344703

[4] Rabin, L. A., Smart, C. M. & Amariglio, R. E. Subjective cognitive decline in preclinical Alzheimer’s disease. *Annu. Rev. Clin. Psychol.* **13**, 369–396 (2017). 28482688 10.1146/annurev-clinpsy-032816-045136

[5] Röhr, S. et al. For cohort studies of memory in an international consortium (COSMIC) Estimating prevalence of subjective cognitive decline in and across international cohort studies of aging: A COSMIC study. *Alzheimers Res. Ther.* **12**, 167 (2020). 33339532 10.1186/s13195-020-00734-y PMC7749505

[6] Rabin, L. A. et al. Alzheimer’s disease neuroimaging initiative; Canadian longitudinal study on aging; health and aging brain study: Health disparities (HABS-HD) study team. Linking self-perceived cognitive functioning questionnaires using item response theory: The subjective cognitive decline initiative. *Neuropsychology* **37**, 463–499 (2023). 37276136 10.1037/neu0000888 PMC10564559

[7] Jester, D. J. et al. Progression from subjective cognitive decline to mild cognitive impairment or dementia: The role of baseline cognitive performance. *J. Alzheimers Dis.* **86**, 1763–1774 (2022). 35253751 10.3233/JAD-215291

[8] Langhough Koscik, R. et al. Validity evidence for the research category, “Cognitively Unimpaired—Declining,” as a Risk marker for mild cognitive impairment and Alzheimer’s disease. *Front. Aging Neurosci.* **13**, 688478 (2021). 34381351 10.3389/fnagi.2021.688478 PMC8350058

[9] Pike, K. E., Cavuoto, M. G., Li, L., Wright, B. J. & Kinsella, G. J. Subjective cognitive decline: Level of risk for future dementia and mild cognitive impairment, a meta-analysis of longitudinal studies. *Neuropsychol. Rev.* **32**, 703–735 (2022). 34748154 10.1007/s11065-021-09522-3

[10] Pitti, H. et al. Cerebrovascular damage in subjective cognitive decline: A systematic review and meta-analysis. *Ageing Res. Rev.* **82**, 101757 (2022). 36240992 10.1016/j.arr.2022.101757

[11] Reisberg, B. et al. Psychometric cognitive decline precedes the advent of subjective cognitive decline in the evolution of Alzheimer’s disease. *Dement. Geriatr. Cogn. Disord.* **49**, 16–21 (2020). 32388509 10.1159/000507286 PMC8846443

[12] Slot, R. E. R., Sikkes, S. A. M., Berkhof, J., Brodaty, H., Buckley, R., Cavedo, E. et al; Alzheimer’s Disease Neuroimaging Initiative; DESCRIPA working group; INSIGHT-preAD study group; SCD-I working group; van der Flier WM. Subjective cognitive decline and rates of incident Alzheimer’s disease and non-Alzheimer’s disease dementia. *Alzheimers Dement*. **15**, 465–476 (2019). 10.1016/j.jalz.2018.10.003 PMC6465066 30555032

[13] Wang, X. T. et al. Association of subjective cognitive decline with risk of cognitive impairment and dementia: A systematic review and meta-analysis of prospective longitudinal studies. *J. Prev. Alzheimers Dis.***8**, 277–285 (2021).34101784 10.14283/jpad.2021.27

[14] Benedictus, M. R. et al. White matter hyperintensities relate to clinical progression in subjective cognitive decline. *Stroke* **46**, 2661–2664 (2015). 26173729 10.1161/STROKEAHA.115.009475

[15] Selnes, P. et al. Diffusion tensor imaging surpasses cerebrospinal fluid as predictor of cognitive decline and medial temporal lobe atrophy in subjective cognitive impairment and mild cognitive impairment. *J. Alzheimers Dis.* **33**, 723–736 (2013). 23186987 10.3233/JAD-2012-121603

[16] Yue, L. et al. Prediction of 7-year’s conversion from subjective cognitive decline to mild cognitive impairment. *Hum. Brain. Mapp.* **42**, 192–203 (2021). 33030795 10.1002/hbm.25216 PMC7721238

[17] Scarth, M., Rissanen, I., Scholten, R. J. P. M. & Geerlings, M. I. Biomarkers of Alzheimer’s disease and cerebrovascular lesions and clinical progression in patients with subjective cognitive decline: A systematic review. *J. Alzheimers Dis.* **83**(3), 1089–1111 (2021). 34397412 10.3233/JAD-210218

[18] Wolfsgruber, S., Molinuevo, J. L., Wagner, M., Teunissen, C. E., Rami, L., Coll-Padrós, N.; Euro-SCD working group. Prevalence of abnormal Alzheimer’s disease biomarkers in patients with subjective cognitive decline: cross-sectional comparison of three European memory clinic samples. *Alzheimer’s Res. Ther. ***11**, 8 (2019). 10.1186/s13195-018-0463-y PMC6337830 30654834

[19] Pavisic, I. M. et al. Subjective cognitive complaints at age 70: Associations with amyloid and mental health. *J. Neurol. Neurosurg Psychiatry.* **92**, 1215–1221 (2021). 34035132 10.1136/jnnp-2020-325620 PMC8522456

[20] Stark, M. et al. Relevance of minor neuropsychological deficits in patients with subjective cognitive decline. *Neurology* **101**, e2185–e2196 (2023). 37821235 10.1212/WNL.0000000000207844 PMC10663030

[21] Munro, C. E. et al. Recent contributions to the field of subjective cognitive decline in aging: A literature review. *Alzheimers Dement (Amst).* **15**, e12475 (2023). 37869044 10.1002/dad2.12475 PMC10585124

[22] Desai, R. et al. Affective symptoms and risk of progression to mild cognitive impairment or dementia in subjective cognitive decline: A systematic review and meta-analysis. *Ageing Res. Rev.* **71**, 101419 (2021). 34390850 10.1016/j.arr.2021.101419

[23] Hessen, E. et al. Subjective cognitive impairment is a predominantly benign condition in memory clinic patients followed for 6 years: The Gothenburg-Oslo MCI study. *Dement. Geriatr. Cogn. Dis. Extra.* **7**, 1–14 (2017). 28413412 10.1159/000454676 PMC5346963

[24] Perron, A. et al. In individuals with subjective cognitive decline, age, memory and speed scores at baseline predict progression to cognitive impairment. *Alzheimer Dis. Assoc. Disord.* **36**, 359–361 (2022). 35867966 10.1097/WAD.0000000000000520

[25] Lezak, M. D., Howieson, D. B. & Loring, D. W. *Neuropsychological Assessment* 4th edn. (Oxford University Press, 2004).

[26] Schmidt, M. Rey Auditory Verbal Learning Test: A handbook. Western Psychological Services (1996).

[27] Almkvist, O., Rennie, A., Westman, E., Wallert, J. & Ekman, U. Methods for assessment of Rey Auditory Verbal Learning test performance in memory clinic patients and healthy adults—At the cross-roads of learning theory and clinical utility. *Clin. Neuropsychol.* **12**, 1–15 (2024). 10.1080/13854046.2024.2384616 39135427

[28] Grasby, P. M. et al. Functional mapping of brain areas implicated in auditory–verbal memory function. *Brain* **116**, 1–20 (1993). 8453452 10.1093/brain/116.1.1

[29] Jeneson, A. & Squire, L. R. Working memory, long-term memory, and medial temporal lobe function. *Learn Mem.* **19**, 15–25 (2011). 22180053 10.1101/lm.024018.111 PMC3246590

[30] Witt, J. A. et al. When does conscious memory become dependent on the hippocampus? The role of memory load and the differential relevance of left hippocampal integrity for short- and long-term aspects of verbal memory performance. *Brain Struct. Funct.* **224**, 1599–1607 (2019). 30863886 10.1007/s00429-019-01857-1

[31] Owen, A. M. The role of the lateral frontal cortex in mnemonic processing: The contribution of functional imaging. *Exp. Brain Res.* **133**, 33–43 (2000). 10933208 10.1007/s002210000398

[32] Wager, T. D. & Smith, E. E. Neuroimaging studies of working memory: A meta-analysis. *Cogn. Affect. Behav. Neurosci.* **3**, 255–274 (2003). 15040547 10.3758/cabn.3.4.255

[33] Wendelken, C., Bunge, S. A. & Carter, C. S. Maintaining structured information: An investigation into functions of parietal and lateral prefrontal cortices. *Neuropsychologia* **46**, 665–678 (2008). 18022652 10.1016/j.neuropsychologia.2007.09.015

[34] Putcha, D., Brickhouse, M., Wolk, D. A., Dickerson, B. C.; Alzheimer’s Disease Neuroimaging Initiative. Fractionating the Rey Auditory Verbal Learning Test: Distinct roles of large-scale cortical networks in prodromal Alzheimer’s disease. *Neuropsychologia.* **129**, 83–92 (2019). 10.1016/j.neuropsychologia.2019.03.015 PMC6571167 30930301

[35] Miller, G. A. The magical number seven, plus or minus two: Some limits on our capacity for processing information. *Psychol. Rev.* **1956**(101), 343–352 (1956). 10.1037/0033-295x.101.2.343 8022966

[36] Yun, R. J., Krystal, J. H. & Mathalon, D. H. Working memory overload: Fronto-limbic interactions and effects on subsequent working memory function. *Brain Imaging Behav.* **4**, 96–108 (2010). 20503117 10.1007/s11682-010-9089-9 PMC2854358

[37] Moran, T. P. Anxiety and working memory capacity: A meta-analysis and narrative review. *Psychol. Bull.* **142**, 831–864 (2016). 26963369 10.1037/bul0000051

[38] Breit, M., Scherrer, V., Tucker-Drob, E. M. & Preckel, F. The stability of cognitive abilities: A meta-analytic review of longitudinal studies. *Psychol. Bull.* **150**, 399–439 (2024). 38330347 10.1037/bul0000425 PMC11626988

[39] Winblad, B. et al. Mild cognitive impairment-beyond controversies, towards a consensus: Report of the international working group on mild cognitive impairment. *J. Int. Med.* **2004**(256), 240–246 (2004). 10.1111/j.1365-2796.2004.01380.x 15324367

[40] Ekman, U. et al. The MemClin project: A prospective multi memory clinics study targeting early stages of cognitive impairment. *BMC Geriatr.* **20**, 1–9 (2020). 10.1186/s12877-020-1478-3 PMC7059672 32138686

[41] World Health Organization. The ICD-10 classification of mental and behavioural disorders: Clinical descriptions and diagnostic guidelines (1992).

[42] Scheltens, P. et al. Atrophy of medial temporal lobes on MRI in “probable” Alzheimer’s disease and normal ageing: Diagnostic value and neuropsychological correlates. *J. Neurol. Neurosurg. Psychiatry.* **55**, 967–972 (1992). 1431963 10.1136/jnnp.55.10.967 PMC1015202

[43] Scheltens, P., Pasquier, F., Weerts, J. G., Barkhof, F. & Leys, D. Qualitative assessment of cerebral atrophy on MRI: Inter- and intra-observer reproducibility in dementia and normal aging. *Eur. Neurol.* **37**, 95–99 (1997). 9058064 10.1159/000117417

[44] Fazekas, F., Chawluk, J. B., Alavi, A., Hurtig, H. I. & Zimmerman, R. A. MR signal abnormalities at 1.5 T in Alzheimer’s dementia and normal aging. *AJR Am. J. Roentgenol.* **149**, 351–356 (1987). 3496763 10.2214/ajr.149.2.351

[45] Dakterzada, F. et al. Assessment of the concordance and diagnostic accuracy between Elecsys and Lumipulse fully automated platforms and Innotest. *Front. Aging Neurosci.* **13**, 604119 (2021). 33746733 10.3389/fnagi.2021.604119 PMC7970049

[46] Wechsler, D. *WAIS-IV Svensk Version [Swedish Version]* (Publisher, 2010).

[47] Cherry, B. J., Buckwalter, J. G. & Henderson, V. W. Better preservation of memory span relative to supraspan immediate recall in Alzheimer’s disease. *Neuropsychologia* **2002**(40), 846–852 (2023). 10.1016/s0028-3932(01)00173-7 11900735

[48] Folstein, M. F., Folstein, S. E. & McHugh, P. R. “Mini-mental state”. A practical method for grading the cognitive state of patients for the clinician. *J. Psychiatr. Res.* **12**, 189–198 (1975). 1202204 10.1016/0022-3956(75)90026-6

[49] Burmester, B., Leathem, J. & Merrick, P. Subjective cognitive complaints and objective cognitive function in aging: A systematic review and meta-analysis of recent cross-sectional findings. *Neuropsychol. Rev.* **26**, 376–393 (2016). 27714573 10.1007/s11065-016-9332-2

[50] Gerton, B. K. et al. Shared and distinct neurophysiological components of the digits forward and backward tasks as revealed by functional neuroimaging. *Neuropsychologia* **42**, 1781–1787 (2004). 15351627 10.1016/j.neuropsychologia.2004.04.023

[51] Petersen, S. E. & Posner, M. I. The attention system of the human brain: 20 years after. *Annu. Rev. Neurosci.* **35**, 73–89 (2012). 22524787 10.1146/annurev-neuro-062111-150525 PMC3413263

[52] Posner, M. I., Rothbart, M. K. & Voelker, P. Developing brain networks of attention. *Curr. Opin. Pediatr.* **28**, 720–724 (2016). 27552068 10.1097/MOP.0000000000000413 PMC5257020

[53] Ausén, B., Edman, G., Almkvist, O. & Bogdanovic, N. Self- and informant ratings of personality in mild cognitive impairment, reviewed. *Dement. Geriatr. Cogn. Disord.* **32**, 387–393 (2011). 22301462 10.1159/000330695

[54] Grober, E. et al. Association of stages of objective memory impairment with incident symptomatic cognitive impairment in cognitively normal individuals. *Neurology* **100**(22), e2279–e2289 (2023). 37076305 10.1212/WNL.0000000000207276 PMC10259282

[55] Karanth, S. D. et al. Four common late-life cognitive trajectories patterns associate with replicable underlying neuropathologies. *J. Alzheimers Dis.* **82**, 647–659 (2021). 34057090 10.3233/JAD-210293 PMC8316292

[56] Tucker-Drob, E. M. Cognitive aging and dementia: A life span perspective. *Annu. Rev. Dev. Psychol.* **1**, 177–196 (2019). 34046638 10.1146/annurev-devpsych-121318-085204 PMC8153102

