# Supplementary material for: Supraspan memory performance is impaired in subjective cognitive impairment compared to cognitively unimpaired individuals
Source: Sci Rep. 2025 Jul 2;15:23071. doi: 10.1038/s41598-025-07664-5 (PMC12215460; doi:10.1038/s41598-025-07664-5)
Supplement: Supplementary file 1 — Supplementary Material 1 [file 41598_2025_7664_MOESM1_ESM.docx]

Supplement Table 1. MANCOVA on memory tests (DV) vs group (MCI and SCI) as IV’s and covariates (age, sex and education).

Group (*F*(1,1345)=128.41, *p*<0.001, *η^2^*=0.160)

Age (*F*(1,1345)=30.92, *p*<0.001, *η^2^*=0.022)

Sex (*F*(1,1345)=10.301, *p*<0.001, *η^2^*=0.008)

Education (*F*(1,1345)=29.02, *p*<0.001, *η^2^*=0.021)

Test x Group (*λ*=0.921, *F*(2,1345)=57.67, *p*<0.001, *η^2^*=0.079)

Test x Age (*λ*=0.966, *F*(1,1345)=47.97, *p*<0.001, *η^2^*=0.034)

Test x Sex (*λ*=0.976, *F*(1,1345)=32.77, *p*<0.001, *η^2^*=0.024)

Test x Education (*λ*=0.976, *F*(1,1345)=32.77, *p*<0.001, *η^2^*=0.024)
