# Supplementary material for: Supraspan memory performance is impaired in subjective cognitive impairment compared to cognitively unimpaired individuals
Source: Sci Rep. 2025 Jul 2;15:23071. doi: 10.1038/s41598-025-07664-5 (PMC12215460; doi:10.1038/s41598-025-07664-5)
Supplement: Supplementary file 2 — Supplementary Material 2 [file 41598_2025_7664_MOESM2_ESM.docx]

Table 2 MANCOVA on memory tests (DV) vs group (MCI and SCI) and brain abnormality (MTA, GCA and WMH, separate analyses) as IV’s and covariates (age, sex and education)

Test (*λ*=0.996, *F*(1, 1002)=4.41, *p*=0.036, *η^2^*=0.008)

Group (*F*(1, 508)=58.02, *p*<0.001, *η^2^*=0.103)

MTA pathology ns

Test x Group (*F*(1, 1002)=45.37, *p*<0.001, *η^2^*=0.043)

Test x MTA ns

Test x MTA x Group ns

Test x Age (*p*<0.001, *η^2^*=0.031)

Test x Sex (*p*<0.001, *η^2^*=0.027)

Test x Education ns

Age (*p*<0.001, *η^2^*=0.015)

Sex (*p*<0.001, *η^2^*=0.010)

Education ns
