# Supplementary material for: Supraspan memory performance is impaired in subjective cognitive impairment compared to cognitively unimpaired individuals
Source: Sci Rep. 2025 Jul 2;15:23071. doi: 10.1038/s41598-025-07664-5 (PMC12215460; doi:10.1038/s41598-025-07664-5)
Supplement: Supplementary file 3 — Supplementary Material 3 [file 41598_2025_7664_MOESM3_ESM.docx]

Supplementary Table 3. MANCOVA on memory tests (DV) vs group (MCI and SCI) and CSF biomarkers (Abeta, p-tau and total tau, separate analyses) as IV’s and covariates (age, sex and education).

Test (*λ*=0.994, *F*(1, 508)=3.05, *p*=0.08, *η^2^*=0.006)

Group (*F*(1, 1002)=95.38, *p*<0.001, *η^2^*=0.087)

CSF biomarkers ns

Test x Group (*λ*=0.950, *F*(1, 508)=26.68, *p*<0.001, *η^2^*=0.050

Test x Abeta ns

Abeta x Group (*F*(1, 508)=5.51, *p*=0.019, *η^2^*=0.011

Test x Age (*p*<0.001, *η^2^*=0.031)

Test x Sex (*p*<0.001, *η^2^*=0.027)

Test x Education ns

Age (*p*<0.001, *η^2^*=0.015)

Sex (*p*<0.001, *η^2^*=0.010)

Education ns

Outcome of ANOVA on frequency of brain atrophy (3 categories, see Method section) vs group (MCI and SCI)

MTA (*χ*^2^(1)=24.00, *p*<0.001, *φ*=0.165)

GCA (*χ*^2^(1)=4.40, *p*<0.05, *φ*=0.065)

WMH (*χ*^2^(1)=12.89, *p*<0.001, *φ*=0.112)

Outcome of ANOVA on frequency of CSF biomarkers (binarized, see Method section) and Group (MCI and SCI)

Abeta (*χ*^2^(1)=12.23, *p*<0.001, *φ*=0.152

p-tau (*χ*^2^(1)=17.31, *p*<0.001, *φ*=0.181)

total-tau (*χ*^2^(1)=13.81, *p*<0.001, *φ*=0.162)

Outcome of t-test on difference between CSF cohorts (CSF yes/no)

Age (*t*(1270)=4.39, *p*<0.001, Cohen’s *d*=0.26)

Cognition (RAVL learning) (*t*(1270)=2.28, *p*<0.05, Cohen
